# CP-ABE Based Privacy-Preserving User Profile Matching in Mobile Social Networks

**DOI:** 10.1371/journal.pone.0157933

**Published:** 2016-06-23

**Authors:** Weirong Cui, Chenglie Du, Jinchao Chen

**Affiliations:** School of Computer Science and Engineering, Northwestern Polytechnical University, Xi’an, PR China,710072; University of South Australia, AUSTRALIA

## Abstract

Privacy-preserving profile matching, a challenging task in mobile social networks, is getting more attention in recent years. In this paper, we propose a novel scheme that is based on ciphertext-policy attribute-based encryption to tackle this problem. In our scheme, a user can submit a preference-profile and search for users with matching-profile in decentralized mobile social networks. In this process, no participant’s profile and the submitted preference-profile is exposed. Meanwhile, a secure communication channel can be established between the pair of successfully matched users. In contrast to existing related schemes which are mainly based on the secure multi-party computation, our scheme can provide verifiability (both the initiator and any unmatched user cannot cheat each other to pretend to be matched), and requires few interactions among users. We provide thorough security analysis and performance evaluation on our scheme, and show its advantages in terms of security, efficiency and usability over state-of-the-art schemes.

## Introduction

Discovering and interacting with people within a certain distance according to personal preferences is a crucial service provided by mobile social networks (MSN), which is helping us to stay connected better than ever. Let us imagine the following two scenarios. (1) At the airport, a passenger wants to discover and connect with the nearby passengers who come from the same university. (2) In the hospital, a patient wants to find similar patients according to their disease symptoms and medications for physical or mental support. In MSN, all such requirements can be satisfied by *user profile matching* very quickly and accurately.

Generally speaking, user profile is a set of attributes generated by users to describe themselves for special friending purpose when they join social networks. For example, in the first scenario, the user profile of a passenger may include his/her age, sex, university from which he/she graduated, company in which he/she is working and his/her destination, etc. In the second scenario, the user profile of a patient may include his/her disease symptoms, medications being taken and his/her doctor, etc. The meaning of “matching” can be defined from different perspectives. For instance, if we consider the user profile as an attribute set, two profiles are matched when the number of their common attributes exceeds a certain threshold. If we consider the user profile as an attribute vector, the meaning of “matching” can be defined as that the distance of the two attribute vectors is less than a certain threshold.

Nowadays, a lot of exciting mobile applications based on user profile matching have been generated. Small-talks [1] connects proximate users based on their common interests. Color [2] allows people in close proximity (within 50 meters) to share photos automatically based on their similarity. MagnetU [3] can automatically match one with nearby users who have the similar profile.

However, such systems also raise a number of privacy concerns. Obviously, directly exchanging the user profiles without any protection may open a door to a variety of attacks. Let us take the aforementioned two scenarios for example. In the first scenario, if an adversary can access the preference-profile submitted by the initiator, he/she may pretend to be the matched one, make contact with the initiator and implement further deception. In the second scenario, if the profiles of patients are directly exchanged with each other, it will facilitate user profiling where that sensitive and private information can be easily collected by a nearby user, either in an active or passive way.

Therefore, the demand for privacy protection motivates people to find *privacy-preserving profile matching* schemes which make the friending in MSN more secure. The most direct solution is to take advantage of a trusted central server to collect information from individual users, compute and disseminate the matching results on demand [4][5][6]. However, this solution may be unsuitable for MSN. First, it requires the user to access the mobile Internet. But, in practice the mobile Internet may not always be available, and it may incur a high expense. Second, the central server may become a bottleneck when a failure or attack occurs. For these reasons, the distributed solution in which users are able to well protect their personal information without the help of a trusted central server is more suitable for MSN, especially for MSN based on short-range wireless technologies such as WiFi and Bluetooth [7][8].

Several distributed solutions of the privacy-preserving profile matching problem have been proposed in recent years. They are mainly based on *secure multi-party computation* (SMC), and can be divided into two mainstreams. The first category treats user profile as attribute set and computes the similarity between two users based on *private set intersection* (PSC) and *private cardinality of set intersection* (PSCI) [9][10]. The second category describes user profile as an attribute vector and measures the similarity by *secure dot-product computation*[11][12][13]. These methods rely on public-key cryptosystem and homomorphic encryption, which results in expensive computation cost. Multiple rounds of interactions are required to perform the presetting and profile matching between each pair of users, which means higher communication cost. In these methods, both matched users and unmatched users are required to take on nearly the same expensive computation and communication cost. Furthermore, most of these methods are unverifiable, which means some malicious adversaries may forge their profiles or return the false calculation results to deceive the initiator.

In this paper, we propose a novel privacy-preserving profile matching scheme which can overcome the above disadvantages. Our scheme is based on ciphertext-policy attribute-based encryption (CP-ABE). Its basic idea is simple. When an initiator wants to search for nearby users who meet his/her preference, he/she generates a ciphertext using CP-ABE at first. His/her preference-profile is taken as the ciphertext access policy and embedded into the ciphertext. This ciphertext is sent to nearby users. Only the matched user whose profile includes the preference-profile can decrypt the ciphertext correctly. Then he/she can choose to communicate with the initiator.

Despite a simple solution, there are two main challenges that should be carefully addressed.

First, there are a lot of CP-ABE constructions, but most of them cannot be applied to our scheme. The most important reason is that the ciphertext access policies in these constructions are accessible to anyone. However, in our application context, the ciphertext access policy (preference-profile) should be hidden for privacy-preserving. In addition, in most CP-ABE constructions, both the size of ciphertext and decryption time increase linearly with the number of user attributes, which cannot satisfy the requirements in terms of communication and computation efficiency in our application context. Therefore, considering the security and efficiency, we have to find out a CP-ABE construction that provides receiver anonymity through hidden access policy while ensuring the constant size of ciphertext and constant decryption time. Facing this challenge, in our work, a special CP-ABE construction [14] with hidden access policy, constant length of ciphertext and constant decryption time is chosen as the foundation of our privacy-preserving profile matching scheme. It builds on the prime order group, and relies on asymmetric *decision bilinear diffie-hellman* (DBDH) assumption. It was shown to be fully secure with respect to the standard model.

Second, in the CP-ABE constructions applied in our work, the size of access policy is required to be equal to the size of user attributes. However, in our application context, the length of the preference-profile is not necessarily equal to the length of profile of matching user. In fact, the meaning of “matching” in our scheme is “including”. That is to say, the profile of the matched user must include all the attributes in the preference-profile. Therefore, we have to design a mechanism to enable the original CP-ABE construction to satisfy such a requirement. In order to overcome this barrier, we modify the original key generation algorithm, design a data structure named *reminder vector*, and design the corresponding algorithm to achieve fast matching.

In summary, the main contributions of this paper are as follows. We design a CP-ABE based privacy-preserving user profile matching scheme. In addition to the capability of privacy-preserving, our method has the following advantages compared with existing related methods. First, it provides verifiability, which means any participant cannot cheat the initiator with the wrong matching result. Second, it can help the matched pair to establish a secure communication channel, which is ignored by most related works. Moreover, the unmatched user can be ruled out very quickly. Furthermore, only a few interactions between the initiator and matching user are required because the matching user can determine the matching result without the help of the initiator, which is significant for decreasing the computation and communication cost.

The rest of this paper is organized as follows. Section Preliminaries presents some preliminaries of CP-ABE. Section System Model and Problem Definition gives the system model and problem definition. Section Our Design describes the protocol design in detail. Section Security and Performance Analysis presents the security proof and efficiency analysis. The experiment results are shown in section Performance Analysis. The comparison with related works is presented in section Related Works and Comparison. Finally, we conclude our works in section Conclusion.

## Preliminaries

Since the CP-ABE construction is the foundation of our work, we introduce the basic knowledge about it in this section. We first give a brief review of attribute-based encryption. Then we describe the syntax of CP-ABE. Finally, we briefly present how to prove the security of a CP-ABE construction.

### Attribute-Based Encryption

The traditional public-key encryption (PKE) [15] is a powerful mechanism to ensure the confidentiality of data storage and transmission. It can work effectively in the scenario that the user who encrypts the sensitive data knows exactly the identity and public key of the recipient. However, in a large complex distributed network environment users often cannot get the public keys and identities from all the recipients. In such a scenario, it is often imperative that user who encrypts sensitive data establishes a specific access control policy on who can decrypt this data. Such requirement led to the evolution of the identity-based encryption (IBE) [16] and then to the attribute-based encryption (ABE) as pioneered in [17]. Using ABE, user can specify a certain policy based on attributes of the recipients, and only the recipient whose attributes meet the policy can decrypt the ciphertext. Since we always manage people or things or thing by their attributes in real life, the ABE is realistic for the real environment.

There are two flavours of ABE defined, i.e. key-policy attribute-based encryption (KP-ABE) [18] and ciphertext-policy attribute-based encryption (CP-ABE) [19]. In KP-ABE, each ciphertext is associated with a set of attributes, and each user secret key is associated with a threshold access structure (i.e. access policy) on attributes. Decryption is enabled if and only if the ciphertext attribute set satisfies the access structure of the user secret key. In CP-ABE the situation is reversed: attributes are associated with user secret keys and access structures with ciphertexts.

Our solution is based on CP-ABE. Although there are a lot of CP-ABE constructions, most of them cannot be applied to our scheme for the reason mentioned in section Introduction. In this paper, we choose a special CP-ABE construction proposed by Xie Li *et al*. [14]. It can provide receiver anonymity by hiding the access policy. Moreover, the length of ciphertext and decryption time in it are constant. Its details are presented in section Our Design.

### The Syntax of CP-ABE

In general, a CP-ABE construction consists of the following four algorithms.

**Setup**(1^*k*^) → *PK*, *MK*. This algorithm takes the security parameter *k* as input and generates a public key *PK* and a master key *MK*.

**Key Generation**(*MK*, *L*) → *SK*. This algorithm takes the master key *MK* and a set of attributes *L* as input and outputs the user’s private key SK associated with *L*. In other words, the user’s attribute set *L* describes his private key SK and determines his right for decryption.

**Encrypt**(*PK*, *M*, *W*) → *CT*. The user uses public key *PK*, and ciphertext access policy *W* to encrypt message *M*, and outputs the ciphertext *CT*. Only the user whose attributes meet *W* can decrypt *CT* correctly.

**Decrypt**(*PK*, *CT*, *SK*) → *M*. In this algorithm, the user uses public key *PK*, his/her private key *SK*, and his/her own attribute set *L* to decrypt *CT*. He/She can decrypt *CT* correctly when and only when his/her attribute set *L* meets the access policy contained in *CT*.

The details of the CP-ABE construction used in our protocol can be seen in section Our Design.

### Bilinear Maps and Complexity Assumptions

#### Bilinear Maps

We give the definition of bilinear map here.

**Definition 1.** (Bilinear Map). *Assume G*, $\hat{G}$ *and* *G*_*T*_ *are three multiplicative cyclic groups of the same prime order p*. *A bilinear map* $e:G\times \hat{G}\to G_{T}$ *is a deterministic function, which takes as input one element from G,* *and one element from* $\hat{G}$ *, and outputs an element in group* *G*_*T*_ *, which satisfies the following criteria:*

1. *Bilinearity: For all x ∈ G,* $y\in \hat{G}$ *,a*, *b* ∈ *Z*_*p*_, *e*(*x*^*a*^, *y*^*b*^) = *e*(*x*, *y*)^*ab*^.
2. *Non degeneracy:* $e(g,\hat{g})\neq 1$ *where g and* $\hat{g}$ *are generators of* *G* *and* $\hat{G}$.
3. e must be computed efficiently.

If $G=\hat{G}$, we call it a symmetric bilinear map, otherwise it is a asymmetrical bilinear map. Our CP-ABE construction is based on the asymmetrical bilinear map.

#### Decisional Bilinear Diffie-Hellman (DBDH) Assumption

DBDH [20] is a complexity assumption which is a cornerstone of the security of ABE. The security of our CP-ABE construction is based on asymmetric DBDH assumption [21] which is extended from the DBDH assumption to asymmetric bilinear groups.

Assume $e:G\times \hat{G}\to G_{T}$ is a asymmetrical bilinear map, where the order of $G,\hat{G}$ and *G*_*T*_ are all prime *p*. *g* and $\hat{g}$ are the generators of *G* and $\hat{G}$ respectively. Consider such a game: the challenger chooses *a*, *b*, *c* $\in Z_{p}^{*}$ at random and then flips a fair binary coin *β* and outputs the tuple (*g*, *g*^*b*^, *g*^*c*^, $\hat{g}$, ${\hat{g}}^{a}$, ${\hat{g}}^{b}$, *Z*). If *β* = 1, $Z=e{\left(g,\hat{g}\right)}^{abc}$, otherwise, *Z* is randomly chosen from *G*_*T*_. The adversary in this game must then output a guess *β*′ of *β*.

The advantage *ϵ* of a adversary B to win this game is defined as:

$$\begin{array}{c} \epsilon =Pr\left[\beta =\beta^{{}^{\prime }}\right]-\frac{1}{2}. \end{array}$$

The DBDH problem now is considered as a NP problem, which means currently there are no probabilistic polynomial-time algorithms to win such a game with a non-negligible advantage.

**Definition 2**. *The (t,ϵ) asymmetric DBDH assumption holds in* *(* $G,\hat{G},G_{T}$*)* *if there is no* *t-time algorithm can solve it with advantage at least ϵ*.

### Security Definitions of CP-ABE

Now we describe the definition of semantic security against passive adversaries for our CP-ABE. Consider the following challenger-adversary game:

**Setup**: The challenger runs the algorithm **Setup** to generate a public key *PK* and a mast key *MK*, then gives *PK* to the adversary.
**Phase 1**: The adversary is allowed to make a series of queries adaptively for private keys associated with attribute lists *L*_1_, *L*_2_, …, *L*_*i*_, 1 ≤ *i* < *q*. In response, The challenger generates private keys *SK*_1_, *SK*_2_, …, *SK*_*i*_, and sends them to the adversary.
**Challenge**: The adversary submits two equal length massages *M*_0_, *M*_1_ and two access policies *W*_0_, *W*_1_ which are not allowed to be satisfied by any attribute list *L*_*i*_ queried by adversary in phase 1. Then the challenger randomly chooses *r*, *e* ∈ {0,1}, generates the ciphertext *T* = *Encrypt*(*PK*, *M*_*r*_, *W*_*e*_) and sends it to the adversary as challenger ciphertext.
**phase 2**: Phase 1 is repeated with the restriction that the queried attribute lists *L*_*i*+1_, *L*_*i*+2_, …, *L*_*q*_ can not satisfy *W*_0_, *W*_1_.
**Guess**: The adversary submits a guess *r*′ of *r*, and *e*′ of *e* and wins this game if *r*′ = *r* and *e*′ = *e*.

**Definition 3.** *Let ϵ be the advantage of the adversary to win the game mentioned above,* $\epsilon =Pr\left[r=r^{{}^{\prime }}\wedge e^{\prime }=e\right]-\frac{1}{4}$. *A CP-ABE construction with hidden access policy is (t,* *q,* *ϵ)-semantically secure if all* *t*-*time* *adversaries making at most q private key queries have at most an* *ϵ* *in breaking the construction*.

## System Model and Problem Definition

### System Model

#### User Profile

In general, each user in a MSN system has a profile, which is the description of himself/herself. In this paper, the user profile is a set of attributes. Table 1 shows a simple example of the profile of user Bob. The first column shows the attribute category names, and the second column shows the corresponding values. Formally, let A be the attribute space consisting of *n* attribute categories, $A=\{A_{1},A_{2},...,A_{n}\}$. Each $A_{i}\in A$ has *m*_*i*_ candidate values, *A*_*i*_ = {*a*_*i*1_, *a*_*i*2_, …, *a*_*im*_*i*__}. A user profile, which is called attribute list hereafter, is generated by two steps. In step one, choose the attribute categories. Next, choose a specific value for each category. The attribute list of user *u*_*i*_ can be represented as $L_{i}=\left\{l_{i}^{1},l_{i}^{2},...,l_{i}^{r_{i}}\right\}$. Each $l_{i}^{k}$ comes from different attribute categories. That is to say, for $l_{i}^{k_{1}}\in A_{j_{1}}$ and $l_{i}^{k_{2}}\in A_{j_{2}}$, *j*_1_ ≠ *j*_2_ if *k*_1_ ≠ *k*_2_. The number of attributes is not necessary the same for different users. Notably, elements in the attribute list are sorted according to the order of the corresponding attribute categories in A, which is vital for profile matching in our scheme.

**Table 1:** Bob’s profile.

Attribute Category	Attribute Value
Age	22
Sex	male
Blood Type	A
Zodiac Sign	Virgos
Home Town	Xi’an
University	NPU
Profession	teacher
Interest	Basketball

#### Profile Matching

Let *L*_*r*_ be an attribute list sent by an initiator *u*_*r*_ who wants to search for friends in our system, and *L*_*i*_ be the attribute list of the user *u*_*i*_ who is in physical proximity to *u*_*r*_. The profile matching between *u*_*r*_ and *u*_*i*_ can be formally described as a function *matching*:

$$\begin{array}{c} matching\left(u_{r},u_{i}\right)=\left\{\begin{array}{cc} 1, & \text{if}\;L_{r}\subseteq L_{i}\\ 0, & \text{if}\;L_{r}⊈L_{i}. \end{array}\right. \end{array}$$

That is to say, if the query attribute list *L*_*r*_ is a subset of *u*_*i*_’s attribute list *L*_*i*_, then the matching operation will be successful.

#### Our System Model

**Fig 1:**
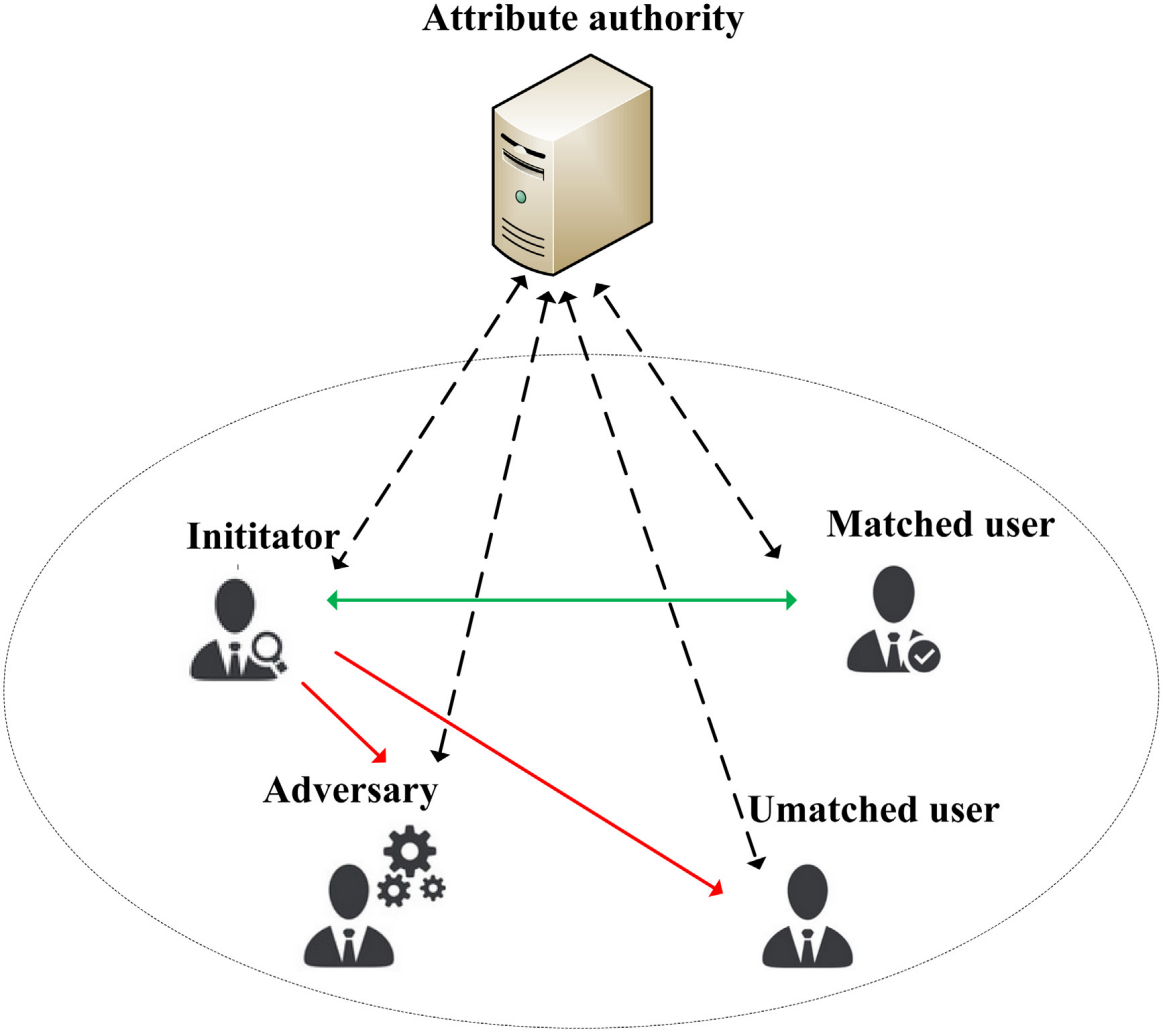
Our System Model.

Fig 1 shows our system model. There are five different roles in it. **Attribute authority** is the trusted central server which can be accessed by all users. *Remarkably*, the access to it only occurs in the initial phase where the user creates his/her own profile, submits registration information and gets the attribute private key. The process of profile matching does not need the server involvement. **Initiator** is the user who sends out the query attribute list. A **matched user** is one whose attribute list matches the initiator’s query attribute list. An **unmatched user** is one whose attribute list cannot match the query attribute list. The **adversary** is a malicious unmatched user who wants to guess the content of query attribute list and even make a further deception. In our system model, we assume that each user controls just one mobile device.

In our system model, users have to download the mobile application based on our protocol from the Internet and set it up on their smart phone. When the application runs for the first time, each user has to create his/her attribute list by choosing attributes from a given attribute space, then submit it to the attribute authority for registration. After that, the attribute authority generates the attribute private key according to the attribute list and sends it together with the public key *PK* back to the user. In addition to that, each user has to generate a pair of keys according to certain predefined public key encryption algorithm.

When an initiator wants to find matched user according to his/her preference in a particular circumstance such as bar, airport or hospital, he/she first generates a query attribute list, and takes it as the access policy to generate a ciphertext which contains his/her own public key. This ciphertext is sent to nearby users. Only the matched user can decrypt the ciphertext correctly. Then, the matched user can choose to communicates with the initiator, and send back a shared communication key encrypted by the initiator’s public key. Remarkably, this process do not need any help of the attribute authority server.

### Adversary Model

In our system, only the matched user is able to learn the content of the query attribute list. If each participant honestly follows our protocol, any unmatched user cannot infer any information from the ciphertext sent by the initiator. However, out of some illegal purpose, the adversary may implement a series of malicious attacks for getting the private information of legitimate users. In this work, we mainly consider the following two powerful malicious attacks.

1. **Dictionary profiling**. The adversary tries to determine the query attribute list by enumerating or guessing all combinations of attributes.
2. **Cheating**. In the process of profile matching, the adversary may cheat by deviating from the agreed protocol, e.g., cheat the initiator with a wrong matching result.

In addition to these two attacks, the adversary may eavesdrop on the conversation between the initiator and the matched user. All these attacks should be well prevented in our scheme.

### Design Goals

1. **Preserving the privacy of user profile**. Only the matched user is able to learn the initiator’s query attribute list. Others cannot infer any information from the query package. Especially, the dictionary profiling attack can be well prevented.
2. **Providing verifiability**. Any user is unable to forge a matching result, which means the cheating attack cannot be implemented successfully.
3. **Providing secure communication channel**. A secure communication channel can be established between the matched pair, which can effectively thwart the eavesdropping attack.
4. **Efficiently filtering**. Since the number of potential friends is usually much smaller than the total number of users in the network, we should efficiently filter out most of the unmatched users so as to speed up the matching process and decrease the computation and communication cost.
5. **Fewer interactions**. Most of the related schemes need multiple rounds of interactions in the profile matching process for privacy-preserving, which causes high computation and communication costs. Therefore, we should try to reduce the number of interactions as much as possible.

## Our Design

### Overview

The main idea of our work is to use the query attribute list *L*_*r*_ as the parameter *W* of the CP-ABE algorithm **Encrypt**(*PK*, *M*, *W*) to encrypt a message, and sent out to users in physical proximity. Only the user whose attribute list *L* matches *L*_*r*_ (*L*_*r*_ ⊆ *L*) can decrypt the message correctly, and then can establish a secure communication channel with the initiator by using the information recovered from the encrypted message. The CP-ABE construction adopted in our work has two characteristics. First, it is receiver anonymous, which means the query attribute list (as the access policy) is hidden in a ciphertext and only the matched user can learn its content. Second, both the length of ciphertext and decryption time in it are constant.

**Fig 2:**
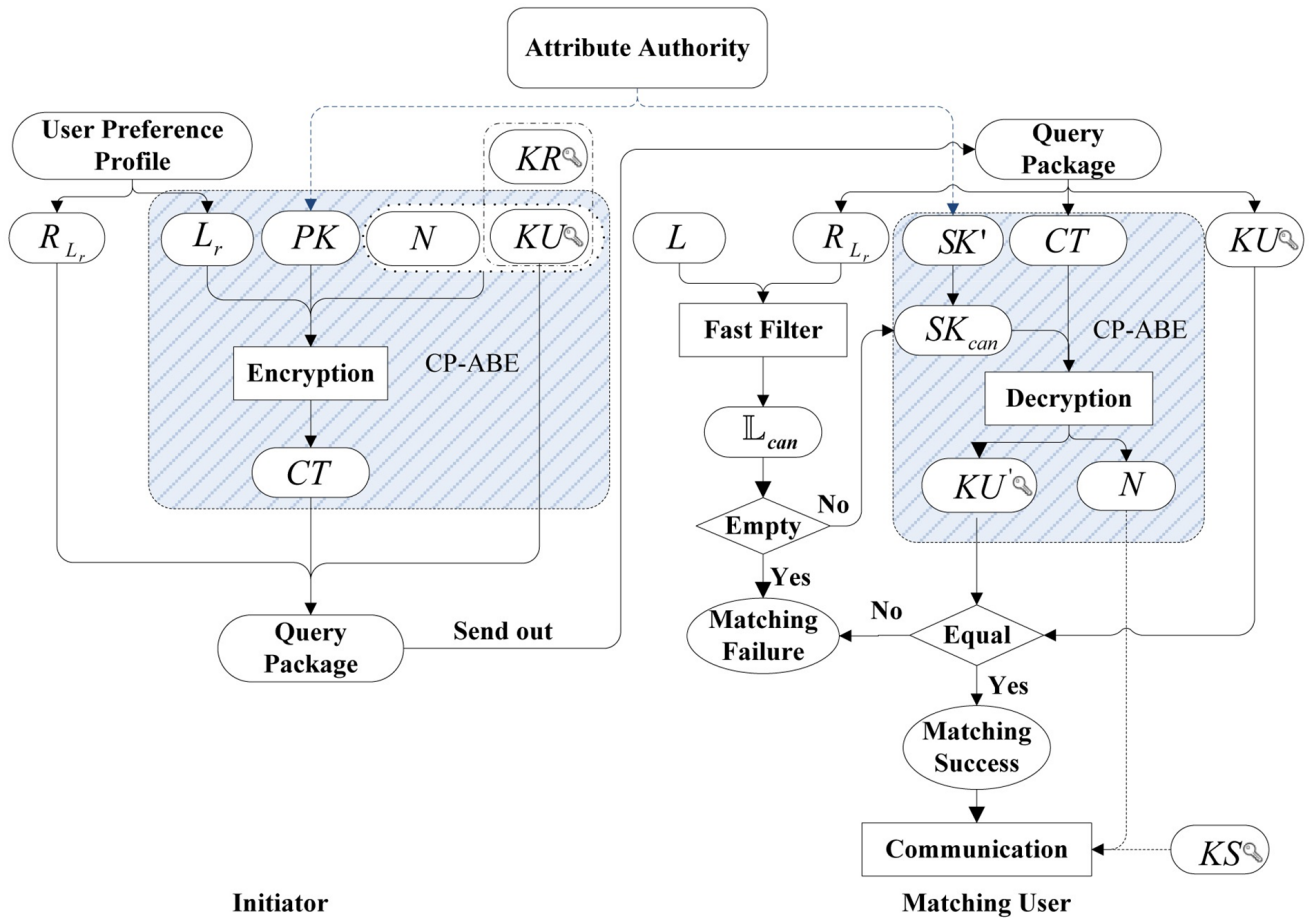
Main process of our scheme.

Fig 2 shows the main process of our privacy-preserving profile matching scheme. For easy understanding, Table 2 lists and explains the notations used in Fig 2. Initially, each user gets *PK* and *SK*′ from the *attribute authority*. Here, *SK*′ is the user’s attribute private key, which is generated and assigned by the attribute authority according to user’s attribute list *L*. Meanwhile, a pair of keys *KU* (public key) and *KR* (private key) are generated by themselves according to the same public key encryption algorithm such as RSA. This key pair will be used for establishing a secure communication channel between the matched users.

**Table 2:** Table of notations.

*L*_*r*_	*Query attribute list*, a set of ordered attributes, used for describing the initiator’s preference profile.
*R*_*L*_*r*__	*Reminder vector*, generated according to *L*_*r*_ and used for fast filtering.
*PK*	*Attribute public key*, generated by attribute authority and used by initiator for encrypting message according to CP-ABE scheme.
*KU*, *KR*	*User key pair*. *KU* (public key) and *KR* (private key) is a pair of keys generated by each user according to certain public key encryption algorithm such as RSA. They are used for establishing a secure communication channel between the matched users.
*N*	*Temporary interactive number*. *N* is a random number generated by initiator. It is used for authenticated key exchange when the match is successful.
*CT*	*Ciphertext*, generated by encrypting *KU* and *N* according to CP-ABE scheme.
*L*	*Attribute list*, a set of ordered attributes used for describing user’s profile.
$L_{can}$	*Candidate attribute list set*. It is the result of fast filter. Each component of $L_{can}$ is an subset of *L* (called candidate attribute list) which is potentially equal to *L*_*r*_.
*SK*′	*User’s attribute private key*, generated and assigned by attribute authority according to user’s attribute list *L*.
*SK*_*can*_	*Candidate user’s private key*. The matching user generates a set of *SK*_*can*_ according to $L_{can}$ and *SK*′. Only one *SK*_*can*_ can decrypt the *CT* correctly.
*KS*	*Shared communication key*, generated by the matched user. The initiator and the matched user can use it for a further secure communication.

The *initiator* starts the matching process by creating a *preference-profile*. Then, it is converted to a *query attribute list* *L*_*r*_ and a *reminder vector* *R*_*L*_*r*__ which is used for fast filtering in the following steps. After that, the *initiator* generates a random number *N* which will be used for authenticated key exchange when the match is successful. Next, the algorithm **Encrypt** using *L*_*r*_ and *PK* as parameters is called to encrypt the message which contains *KU* and *N*. It outputs the ciphertext *CT*. Finally, a *query package*, which contains *R*_*L*_*r*__, *CT* and *KU*, is generated and sent out for profile matching.

When a user in physical proximity to the *initiator* receives the *query package*, he/she first makes a fast filtering computation according to *R*_*L*_*r*__ contained in received message and his/her own *attribute list* *L*, and outputs a set of *candidate attribute lists* $L_{can}$. Each component of $L_{can}$ is an subset of *L* (called candidate attribute list) which is potentially equal to *L*_*r*_. If $L_{can}$ is null, the profile matching fails. Otherwise, using $L_{can}$ and *SK*′ as input, a set of *candidate private keys* are figured out. Each candidate private key *SK*_*can*_ in this set is used by the *matching user* to try to decrypt the ciphertext *CT*. In order to confirm the correctness of the decryption result, *KU*′, which is gained by decrypting *CT*, is compared with *KU*. The decryption result is correct if these two keys are equal, which means the matching successful. After that, the matched user can choose to communicate with the initiator. Specifically, he/she can generate a shared secret key, encrypt it together with *N* using *KU*, and send it back to the initiator. When the initiator gets the shared communication key *KS*, a secure pair-wise secure communication channel can be established between the matched pair by using certain symmetric encryption scheme. The details can be seen in protocol 1.

### The CP-ABE Construction

As we can see in Fig 2, an appropriate CP-ABE construction is the core of our method. Literature [14] provides such a CP-ABE construction, where the ciphertext length is constant and the access policy is hidden. Therefore, we adopt it in our work with a modification in terms of the key generation algorithm. Its formal description is described as follows.

**Setup**(1^*k*^): In the phase of system initialization, the attribute authority chooses two different groups *G*, $\hat{G}$ with the same prime order *p* according to the security parameter 1^*k*^. let $e:G\times \hat{G}\to G_{T}$ denotes the bilinear map and *g*, $\hat{g}$ be the generators of *G*, $\hat{G}$ respectively. Then, the attribute authority chooses secrets $\alpha \in_{R}Z_{p}^{*}$, $\beta \in_{R}Z_{p}^{*}$, $\delta \in_{R}Z_{p}^{*}$ and gets values ${\hat{g}}_{1}={\hat{g}}^{\alpha }$, ${\hat{g}}_{2}={\hat{g}}^{\beta }$, *g*_2_ = *g*^*β*^, $\hat{f}={\hat{g}}^{\delta }$, *f* = *g*^*δ*^. The symbol “∈_*R*_” means “randomly choose from”.

Let A be the attribute space, $A=\{A_{1},A_{2},...,A_{n}\}$. Each $A_{i}\in A$ is a attribute category, which consists of a set of attributes, *A*_*i*_ = {*a*_*i*1_, *a*_*i*2_, …, *a*_*im*_*i*__}. For each attribute *a*_*ij*_ ∈ *A*_*i*_, the attribute authority chooses $\psi_{ij}\in_{R}Z_{p}^{*}$, computes $\Psi_{ij}=g^{\psi_{ij}}$. Let $Y=e(g_{2},{\hat{g}}_{1})$, the public key *PK* is

$$\begin{array}{c} PK=\{g,g_{2},\hat{g},{\hat{g}}_{1},{\hat{g}}_{2},f,Y,\Psi_{ij}\left(1\leq i\leq n,1\leq j\leq m_{i}\right)\}. \end{array}$$

The master private key *MK* is

$$\begin{array}{c} MK=\{{\hat{g}}_{2}^{\alpha },\hat{f},\psi_{ij}\left(1\leq i\leq n,1\leq j\leq m_{i}\right)\}. \end{array}$$

**KeyGeneration**(*MK*, *L*): Let *L* = {*l*_1_, *l*_2_, …, *l*_*q*_} be an attribute list that consists of a set of attributes. Each *l*_*i*_ in *L* comes from a certain attribute category, i.e., *l*_*i*_ ∈ *A*_*k*_. A private key for *L* is generated as follows. First, a random $r\in_{R}Z_{p}^{*}$ is chosen. Then compute

$$D_{0}={\hat{g}}^{r},D_{1}={\hat{g}}_{2}^{\alpha }(\hat{f}\cdot {\hat{g}}^{\sum_{a_{ij}\in L}\psi_{ij}}{)}^{r}.$$

The private key is *SK* = {*D*_0_, *D*_1_}.

Notably, we assume for ∀*L*, *L*′(*L* ≠ *L*′), ∑_*a*_*ij*_ ∈ *L*_ *ψ*_*ij*_ ≠ ∑_*a*_*ij*_ ∈ *L*′_ *ψ*_*ij*_. If there exist *L* and *L*′(*L* ≠ *L*′) such that ∑_*a*_*ij*_ ∈ *L*_ *ψ*_*ij*_ = ∑_*a*_*ij*_ ∈ *L*′_ *ψ*_*ij*_, a user with attribute list *L*′ can decrypt a ciphertext associated with access police *W*, where *L*′ ⊭ *W* and *L* ⊨ *W*. Remark that the assumption holds with overwhelming probability $\frac{p(p-1)...(p-N-1)}{p^{N}}>\frac{{\left(p-(N-1)\right)}^{N}}{p^{N}}={\left(1-\frac{N-1}{p}\right)}^{N}>1-\frac{N(N-1)}{p}>1-\frac{N^{2}}{p}$, where $N=\sum_{i=1}^{n}\left|A_{i}\right|$. Therefore, if each secret key *ψ*_*ij*_ is chosen at random from $Z_{p}^{*}$, then our assumption is natural [22].

However, this key generation algorithm cannot be applied in our setting directly. In order to illustrate the reason, consider the following example. Assume the attribute list of the user *u* is *L* = {*a*_1,2_, *a*_2,6_, *a*_3,3_, *a*_4,7_, *a*_5,1_}, *a*_*ij*_ ∈ *A*_*i*_. Now assume the query attribute list is *L*_*r*_ = {*a*_1,2_, *a*_3,3_}. Given a ciphertext encrypted by *L*_*r*_, although *L*_*r*_ ⊂ *L*, the user *u* cannot decrypt the ciphertext by *SK*. The reason is ∑_*a*_*ij*_ ∈ *L*_*r*__ *ψ*_*ij*_ ≠ ∑_*a*_*ij*_ ∈ *L*_ *ψ*_*ij*_. In order to address this problem, we modify the original key generation algorithm. We decompose the original algorithm **KeyGeneration** into two steps which run on the attribute authority side and the user side respectively. These two steps are:

1. **KeyGenA**(*MK*, *L*)→*SK*′. This algorithm runs on the attribute authority side, and its input parameters is the same as that of the original algorithm **KeyGeneration**. It outputs a private key *SK*′ containing three parts. $SK^{{}^{\prime }}=\left\{D_{0},D_{1}^{{}^{\prime }},{\left\{\hat{D_{k}}\right\}}_{\left(1\leq k\leq |L|\right)}\right\}$. In that,
  $$\begin{array}{c} D_{0}={\hat{g}}^{r},D_{1}^{{}^{\prime }}={\hat{g}}_{2}^{a}\cdot {\hat{f}}^{r},\hat{D_{k}}={\hat{g}}^{r\cdot \psi_{ij}},\left(l_{k}=a_{ij}\right). \end{array}$$
2. **KeyGenU**(*SK*′, *L*_*can*_)→*SK*_*can*_. This algorithm runs on the user side. It takes the output of **keyGenA** and a candidate attribute list *L*_*can*_ = {*l*_*k*_1__, *l*_*k*_2__, …, *l*_*k*_*p*__}(*L*_*can*_ ⊆ *L*) as input, outputs the candidate private key *SK*_*can*_ which contains two components. *SK*_*can*_ = {*D*_0_, *D*_*can*_}. In that,
  $$D_{0}={\hat{g}}^{r},D_{can}=D_{1}^{\prime }\;\prod_{1\leq i\leq p}\hat{D_{k_{i}}}={\hat{g}}_{2}^{a}(\hat{f}\cdot {\hat{g}}^{\sum_{a_{ij}\in L_{can}}\psi_{ij}}{)}^{r}.$$

Now let us continue to consider the example. According to our modified algorithm, $SK^{\prime }=\left\{D_{0},D_{1}^{{}^{\prime }},\hat{D_{1}},\hat{D_{2}},\hat{D_{3}},\hat{D_{4}},\hat{D_{5}}\right\}$, $\hat{D_{1}}={\hat{g}}^{r\cdot \psi_{1,2}}$, $\hat{D_{2}}={\hat{g}}^{r\cdot \psi_{2,6}}$, $\hat{D_{3}}={\hat{g}}^{r\cdot \psi_{3,3}}$, $\hat{D_{4}}={\hat{g}}^{r\cdot \psi_{4,7}}$, $\hat{D_{5}}={\hat{g}}^{r\cdot \psi_{5,1}}$. When the user *u* chose *L*_*can*_ = {*a*_1,2_, *a*_3,3_} as the candidate attribute list according to the hint provide by the reminder vector, he can use *D*_0_, $D_{1}^{{}^{\prime }}$, $\hat{D_{1}}$, $\hat{D_{3}}$ to generate the candidate key.

$$SK_{can}=\{D_{0},D_{can}\},D_{0}={\hat{g}}^{r},D_{can}=D_{1}^{\prime }\cdot \hat{D_{1}}\cdot \hat{D_{3}}={\hat{g}}_{2}^{a}(\hat{f}\cdot {\hat{g}}^{\sum_{a_{ij}\in L_{can}}\psi_{ij}}{)}^{r}.$$

Here, ∑_*a*_*ij*_ ∈ *L*_*r*__ *ψ*_*ij*_ = ∑_*a*_*ij*_ ∈ *L*_*can*__ *ψ*_*ij*_. Therefore, *SK*_*can*_ is able to be used to decrypt the ciphertext correctly.

**Encrypt**(*PK*, *M*, *W*): Parameter *W* is the access policy that determines who can decrypt the ciphertext. Let *W* = {*w*_1_, *w*_2_, …, *w*_*t*_}. Each *w*_*i*_ in *W* comes from a certain attribute category, i.e., *w*_*i*_ ∈ *A*_*k*_. For message *M* ∈ *G*_*T*_, the encryptor chooses $s\in_{R}Z_{p}^{*}$, then computes $\hat{C},C_{0},C_{1}$ as follows:

$$\begin{array}{c} \hat{C}=M\cdot Y^{s},C_{0}=g^{s},C_{1}={\left(f\prod_{a_{ij}\in W}\Psi_{ij}\right)}^{s}. \end{array}$$

The algorithm outputs the ciphertext $CT=\{\hat{C},C_{0},C_{1}\}$.

**Decrypt**(*PK*, *CT*, *SK*): In this algorithm, a decryptor computes $M=\hat{C}e\left(C_{1},D_{0}\right)/e\left(C_{0},D_{1}\right)$ (This computation is based on the original KeyGeneration algorithm. If the input parameter is *SK*_*can*_, the ciphertext is computed as $CT=\hat{C}e\left(C_{1},D_{0}\right)/e\left(C_{0},D_{can}\right)$). Its correctness is shown as follows:

$$\begin{array}{ll} \hat{C}\frac{e(C_{1},D_{0})}{e(C_{0},D_{1})} & =Me{\left(g,\hat{g}\right)}^{\alpha \beta s}\frac{e{\left(f\prod_{a_{ij}\in W}\Psi_{ij},\hat{g}\right)}^{rs}}{e(g^{s},{\hat{g}}_{2}^{a}(\hat{f}{\hat{g}}^{\sum_{a_{ij}\in L}\psi_{ij}}{)}^{r})}\\ & =Me{\left(g,\hat{g}\right)}^{\alpha \beta s}\frac{e(g^{\delta +\sum_{a_{ij}\in W}\psi_{ij}},\hat{g}{)}^{rs}}{e{\left(g,\hat{g}\right)}^{\alpha \beta s}e(g,{\hat{g}}^{\delta +\sum_{a_{ij}\in L}\psi_{ij}}{)}^{rs}}\\ & =M. \end{array}$$

If the bilinear map $e:G\times \hat{G}\to G_{T}$ is symmetrical, which means $G=\hat{G}$, the adversary can guess the access policy *W* by DDH test. That is to say, the adversary randomly chooses an access policy *W*′, computes *e*(*C*_0_, *f*∏_*a*_*ij*_ ∈ *W*′_ *Ψ*_*ij*_) = *e*(*g*^*s*^, *f*∏_*a*_*ij*_ ∈ *W*′_ *Ψ*_*ij*_), then determines whether it equals *e*(*g*, *C*_1_) = *e*(*g*, (*f*∏_*a*_*ij*_ ∈ *L*_ *Ψ*_*ij*_)^*s*^). The adversary can repeat such a computation until he finds the *W*′ which may be equal to *W*. However, in our scheme, $G\neq \hat{G}$, and the adversary cannot get ${\hat{g}}^{s}$. Therefore, the adversary cannot implement the DDH test mentioned here to guess the access policy *W*. That is why our CP-ABE scheme is able to hide the access policy and achieve receiver anonymity.

### Filtering Out The Unmatched User

Obviously, the number of potential matched users is usually much smaller than the total number of users in a network. Based on this fact, we design a mechanism to fast filter out the unmatched user so as to improve the profile matching efficiency. The key data structure of this mechanism is the reminder vector, the components of which are reminders of the hash values of the corresponding attributes in the attribute list divided by *λ*. Such a reminder vector is sent to other users together with the query attribute list by the initiator. For users involved in matching, the first step after receiving the query package is to determine if he/she is the potential target of the initiator by comparing his/her own attribute list with the reminder vector. If not, the profile matching process ends. Otherwise, the further matching computation is implemented.

#### Reminder Vector

Assume that there are *q* attributes in the query attribute list $L_{r}=\left\{l_{r}^{1},l_{r}^{2},...,l_{r}^{q}\right\}$. *λ* is a prime larger than *q*. Let *Hash* be a cryptographic hash function which yields *n*-bit length hash values. A reminder vector *R*_*L*_*r*__ consists of the reminders of all hash values of attributes in *L*_*r*_ divided by *λ*. For each *l*_*r*_ ∈ *L*_*R*_, Let $r_{k}\equiv Hash\left(l_{r}^{k}\right)\;mod\;\lambda$, then *R*_*L*_*r*__ = {*r*_1_, *r*_2_, …, *r*_*q*_}. The following theorem is straightforward.

**Theorem 1** *Consider two attributes* *l*^*i*^, *l*^*j*^, *reminder* *r*^*i*^ ≡ *Hash*(*l*^*i*^) *mod* *λ*, *r*^*j*^ ≡ *Hash*(*l*^*j*^) *mod* *λ*, if *r*^*i*^ ≠ *r*^*j*^, then *l*^*i*^ ≠ *l*^*j*^.

#### The Fast Filtering Algorithm

Algorithm 1 describes how to rule out the unmatched user quickly. It takes a reminder vector *R*, a user attribute list *L* and a prime *λ* as inputs, and outputs a candidate attribute list set $L_{can}$. If $L_{can}=\emptyset$, it means the user’s attribute list cannot match the query attribute list.

In our system model, the number of attributes is not necessary the same for different users. According to our definition of “matching”, for any potential matched user, the length of his/her attribute list must be greater than the length of the query attribute list. Lines 1 to 3 in algorithm 1 does such a check to rule out the users whose attribute list length cannot satisfy this requirement. In lines 4 to 14, for each *r*_*i*_ ∈ *R*, an attribute set *H*_*i*_ is constructed. Here ∀*l*_*j*_ ∈ *H*_*i*_, *r*_*i*_ ≡ *Hash*(*l*_*j*_) *mod* *λ*. In other words, let $l_{r}^{i}\in L_{r}$ be the corresponding attribute of *r*_*i*_ ∈ *R*, each *l*_*j*_ ∈ *H*_*i*_ is likely to be equal to $l_{r}^{k}$ according to theorem 1. After that, if any $H_{i}\in H$ is ∅, which means there is no equivalent attribute *l*_*i*_ ∈ *L* for the corresponding attribute $l_{r}^{k}\in L_{r}$, the user should be filtered out (lines 15 to 17). A candidate attributes list *L*_*can*_ is a combination of one element from each $H_{i}\in H$. For example, choosing *l*_*k*_1__ from *H*_1_, *l*_*k*_2__ from *H*_2_, …, *l*_*k*_*q*__ from *H*_*q*_, *L*_*can*_ = {*l*_*k*_1__, *l*_*k*_2__, …, *l*_*k*_*q*__} is such an candidate attribute list. Remarkably, In our system model, elements in an attribute list are sorted according to the index of their corresponding attribute categories in A. Therefore a legal attribute list *L*_*can*_ should be ordered. This requirement can be formally described as follows:

$$\begin{array}{c} \forall l_{x},l_{y}\in L_{can},l_{x}\in H_{i},l_{y}\in H_{j},i<j\Rightarrow x<y. \end{array}$$

In line 18, the function **Combine** (as shown in Algorithm 2) figures out a set $L_{can}$ which consists of all possible candidate attribute lists.

**Algorithm 1** FastFilter

**Input:**

 1. Reminder vector *R* = [*r*_1_, *r*_2_, …, *r*_*q*_]^*T*^

 2. User attribute list *L* = {*l*_*l*_, *l*_2_, …, *l*_*p*_}

 3. Prime *λ*

**Output:**

 1. The candidate attribute list set

  $L_{can}=\left\{L_{can}^{1},L_{can}^{2},..,L_{can}^{t}\right\}$ ($L_{can}^{i}=\left\{l_{k_{1}},l_{k_{2}},...,l_{k_{q}}\right\}$)

1: **if** |*R*|>|*L*| **then**

2:  **return** ∅

3: **else**

4:  $H=\{H_{1},H_{2},...,H_{q}\}$

5:  **for each** $H_{i}\in H$ **do**

6:   *H*_*i*_ = ∅

7:  **end for**

8:  **for each** *r*_*i*_ ∈ *R* **do**

9:   **for each** *l*_*j*_ ∈ *L* **do**

10:    **if** *r*_*i*_ = *Hash*(*l*_*j*_) *mod* *λ* **then**

11:     *H*_*i*_ = *H*_*i*_∪*l*_*j*_

12:    **end if**

13:   **end for**

14:  **end for**

15:  **if** $\exists H_{i}\in H=\emptyset$ **then**

16:   **return** ∅

17:  **else**

18:   $L_{can}$ = Combine(H)

19:   **return** $L_{can}$

20:  **end if**

21: **end if**

**Algorithm 2** Combine

**Input:**

 1. $H=\{H_{1},H_{2},...,H_{q}\}$

**Output:**

 1.  $L_{can}=\left\{L_{can}^{1},L_{can}^{2},...,L_{can}^{t}\right\}$

1: **if** |H|=1 **then**

2:  $L_{can}=\emptyset$

3:  **for each** $l_{k}\in H\left[1\right]$ **do**

4:   $L_{can}=L_{can}\cup \left\{l_{k}\right\}$

5:  **end for**

6:  **return** $L_{can}$

7: **else**

8:  *H* = Pop(H)

9:  K = Combine(H)

10:  **if** K=∅ **then**

11:   **return** ∅

12:  **else**

13:   $L_{can}=\emptyset$

14:   **for each** $L_{can}^{k}\in K$ **do**

15:    **for each** *l*_*i*_ ∈ *H* **do**

16:     **if** Index(*l*_*i*_) < Index($L_{can}^{k}\left[1\right]$) **then**

17:      $C=\left\{l_{i}\right\}\cup L_{can}^{k}$

18:      $L_{can}=L_{can}\cup C$

19:     **end if**

20:    **end for**

21:   **end for**

22:   **return** $L_{can}$

23:  **end if**

24: **end if**

### Decryption and Confirmation

In order to determine if there exists a candidate attribute list *L*_*can*_ in $L_{can}$ that is equal to the query attribute list *L*_*r*_ (i.e., the user attribute list *L*_*u*_ matches the query attribute list *L*_*r*_), for each *L*_*can*_, we first use it to generate a candidate private key *SK*_*can*_, then try to decrypt the ciphertext by it. The decryption will return a tuple which contains *KU*′ and *N*. If *KU*′ is equal to *KU*, the decryption result is correct, which means the profile match is successful. Then *N* is returned for subsequent steps.

### Secure Communication Channel Establishment

**Fig 3:**
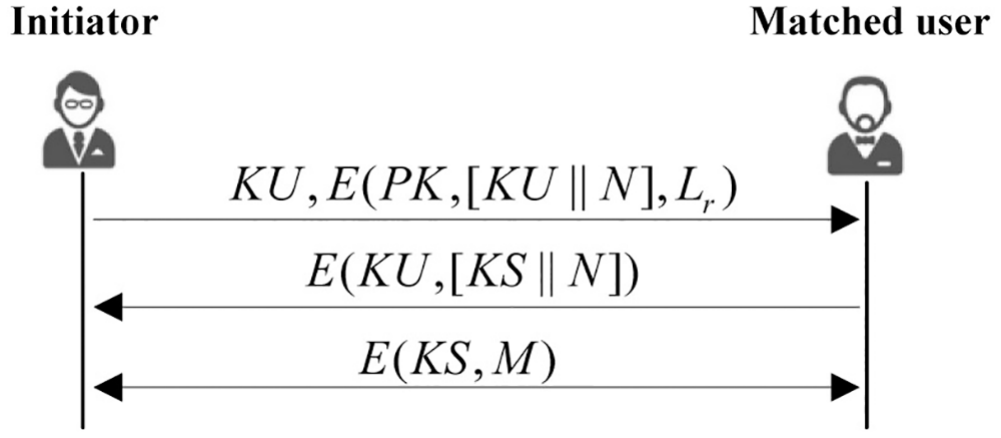
The key exchange process.

If the match process is successful, the matched user can choose to respond to the request. First he/she generates a shared communication key *KS*. Then he/she encrypts *KS* and *N* using initiator’s public key *KU*, and send it back to the initiator. When the initiator gets the reply, he/she decrypts it by using his/her private key *KR*. By authenticating *N*, the initiator can confirm that this reply comes from the user whose attribute list matches the query attribute list, and establish a secure communication channel with the matched user by *KS*. This process can be seen in Fig 3.

**Algorithm 3** CipherDecryption

**Input:**

 1. Ciphertext *CT*

 2. Attribute Public Key *PK*

 3. Attribute Private Key *SK*′(generated by **KeyGenA**(*MK*, *L*))

 4. Candidate Attribute Lists Set $L_{can}$

 5. Initiator’s Public Key *KU*

**Output:**

 1. Temporary Interactive Number *N*

1: **if** $L_{can}=\emptyset$ **then**

2:  **return** NULL

3: **end if**

4: **for each** $L_{can}^{i}\in L_{can}$ **do**

5:   *SK*_*can*_ = **KeyGenU**($SK^{\prime },L_{can}^{i}$)

6:   (*KU*′, *N*) = **Decrypt**(*PK*, *CT*, *SK*_*can*_)

7:   **if** *KU*′ = *KU* **then**

8:    **return** *N*

9:   **else**

10:    **return** NULL

11:   **end if**

12: **end for**

### The Complete Privacy-Preserving Profile Matching Protocol

In this subsection, we describe the complete privacy-preserving profile matching protocol proposed in this paper. As we can see in protocol 1, the complete protocol consists of three phases. Remarkably, the communication between a user and the attribute authority only occurs in the setup phase. Once the communication is over, the user will no longer communicate with the sever unless special circumstances occur. In other words, the communication between a user and the attribute authority occurs only when the user generates or modifies his/her attributes list. Therefore, the profile matching operation among users is completely distributed.

**Protocal 1** The Privacy-Preserving Profile Matching Protocol

**Setup Phase**

 1. The attribute authority A runs **Setup**(1*^k^*) to generate *PK*, *MK*.

 2. Every participant *u*_*i*_ generates his/her attribute list *L*_*u*_*i*__ based on attribute space A.

 3. Every participant *u*_*i*_ submits *L*_*u*_*i*__ to A, and A runs **KeyGenA**(*MK*, *L*_*u*_*i*__) to generate $SK_{u_{i}}^{{}^{\prime }}$, and send back *PK*, $SK_{u_{i}}^{{}^{\prime }}$ to *u*_*i*_.

 4. Every participant *u*_*i*_ generates a key pair (*KU*, *KR*) according to a certain public key encryption algorithm.

**Profile Matching Phase**

 1. The initiator *u*_*a*_ generates query attribute list *L*_*r*_ to describe the user whom he wants to match.

 2. *u*_*a*_ generates the reminder vector *R*_*L*_*r*__ (mod *λ*) for *L*_*r*_.

 3. *u*_*a*_ generates a random number *N*.

 4. *u*_*a*_ runs **Encrypt**(*PK*, *M*, *L*_*r*_) and generates the ciphertext *CT*. *M* = *KU*||*N*.

 5. *u*_*a*_ sends out the tuple (*R*_*L*_*r*__, *KU*, *CT*).

 6. After receiving (*R*_*L*_*r*__, *KU*,*CT*), the user *u*_*b*_ takes *R*_*L*_*r*__, *L*_*u*_*b*__, and prime number *λ* as the inputs of algorithm 1 (**FastFilter**) to figure out $L_{can}$.

 7. If $L_{can}=\emptyset$, the profile matching process ends. Otherwise, *u*_*b*_ continues to run algorithm 3 (**CipherDecryption**).

**Secure Communication Phase**

 1. If algorithm 3 (**CipherDecryption**) returns *N*, it means that *L*_*u*_*b*__ matches the query attribute list *L*_*r*_. Then *u*_*b*_ generates a shared communication key *KS*, encrypt *KS* and *N* by *KU*, and then sends it back.

 2. *u*_*a*_ decrypts the reply by his/her private key *KR*.

 3. If *N* is correct, *u*_*a*_ and *u*_*b*_ make a further secure communication by *KS* using certain predefined symmetric cryptographic technique.

## Security and Performance Analysis

### Security Analysis

Let *PS*(*L*_*r*_, *u*_*i*_) be a decision function. If a user *u*_*i*_ can learn the content of the query attribute list *L*_*r*_, *PS*(*L*_*r*_, *u*_*i*_) = 1; if not, *PS*(*L*_*r*_, *u*_*i*_) = 0. The goal of our scheme is: only for *u*_*i*_ whose attribute list can match *L*_*r*_, *PS*(*L*_*r*_, *u*_*i*_) = 1.

The security of this CP-ABE construction adopted in our scheme provides the fundamental assurance of *privacy-preserving*. In order to achieve subset matching and improve the matching efficiency, we design the reminder vector and corresponding algorithm to achieve fast matching. However, such a efficiency improving design reduces the privacy security because the reminder vector reveals some information about the query attribute list. Theoretically, an adversary may take advantage of the reminder vector to implement dictionary profiling attack. In this subsection, we first present the security proof of the CP-ABE construction. Then we illustrate that our scheme can prevent the dictionary profiling attack by means of quantitative and qualitative analysis. Finally we show the verifiability and communication security of our scheme.

#### The CP-ABE Security

The security of the CP-ABE construction is the cornerstone of the entire system security proposed in this paper. In this subsection, we show that the CP-ABE construction adopted in this paper can provide ANON-IND-CPA security, which means the adversary cannot infer any information about the message *M* and the access policy *W* from the ciphertext. Formally, we give the following theorem.

**Theorem 2.** *The CP-ABE scheme in our paper is* *(t*, *q*, *ϵ) secure assuming the asymmetric decisional (t*+*O*(*ϵ*^−2^ln(*ϵ*^−1^)*λ*^−1^ln(*λ*^−1^)), $\frac{\epsilon }{32(n+1)q}$) *BDH assumption holds, where* $\lambda =\frac{1}{8(n+1)q}$.

*proof*. Suppose there exists a (*t*, *q*, *ϵ*)-adversary A who tries to attack our CP-ABE construction. We construct a simulator B to play the asymmetric DBDH game. Choose $a\in_{R}Z_{p}^{*}$, $b\in_{R}Z_{p}^{*}$, $c\in_{R}Z_{p}^{*}$. Let *g*, $\hat{g}$ be the generators of *G*, $\hat{G}$ respectively. A fair binary coin *β* is fliped. If *β* = 1, then choose $Z=e{\left(g,\hat{g}\right)}^{abc}$, otherwise, choose *Z* randomly from *G*^*T*^. Give the tuple $\left(g,g^{b},g^{c},\hat{g},{\hat{g}}^{a},{\hat{g}}^{b},Z\right)$ to the simulator to take asymmetric DBDH challenge. Then simulator B runs A by executing ANON-IND-CPA game, and finally gives out a guess *β*′ of *β*. By constructing such a simulator, the security problem of our CP-ABE is reduced to the asymmetric DBDH problem. Since the asymmetric DBDH problem is assumed to be NP hard, our CP-ABE construction provides ANON-IND-CPA security. For easy reading, we put the construction details of the simulator and the main proof steps in S1 Appendix.

#### Dictionary Profiling Attack Prevention

Now we consider the *dictionary profiling attack*, which seems to be the most threatening malicious attack against our protocol. In our scheme, although the adversary cannot infer any information about the query attribute list from the received ciphertext, he/she may take advantage of the reminder vector to implement dictionary profiling. A successful dictionary profiling attack depends on two steps. First, given a reminder vector, the adversary has to construct candidate attribute lists by enumerating the attribute combinations from attribute space A. The reminder vectors of these candidate attribute lists should be equal to the given reminder vector. Second, the adversary has to communicate with the attribute authority to generate the private keys for each candidate attribute list, and then try to decrypt the ciphertext. In practice, such an attack is hard to work, and it can be effectively prevented in our scheme. In contrast to other existing schemes, the user profile in our scheme cannot be changed locally, which is the key mechanism for dictionary profiling prevention. Assume the number of attribute categories in A is *n*, and each category has *m* attributes averagely. Given a reminder vector *R* with length *q* and prime *λ*, the number of candidate attribute lists generated by adverary will be (nq)⌊(mλ)⌋q. Obviously, this number will be larger if the attribute space A is larger and *λ* is set properly. In order to prevent the adversary from getting private keys for each candidate attribute list from the attribute authority, we can restrict the private key generation frequency for the same user in the attribute authority by some simple methods. Therefore, our scheme can well address the dictionary profiling attack.

#### Verifiability

In our protocol, the initiator uses temporary interactive number *N* to verify the matching results. *N* is a random number and generated by the initiator. Only the matched user can get gain access to *N* by decrypting the query ciphertext. When the initiator receives the reply which comes from the so-called “matched user”, he/she decrypts it using his/her private key *KR* and verifies the *N* contained in the decryption result. If *N* is correct, the initiator can confirm that this reply comes from an authentic matched user. For an unmatched adversary, he/she cannot forge a reply which can pass the verification since he/she cannot get the correct *N*.

#### Communication Security

According to our protocol, the shared communication key *KS* generated by the matched user can be transmitted securely to the initiator, which depends on a specific public key encryption algorithm such as RSA. Then the matched pair can communicate with each other by using *KS*. Therefore, the adversary who is eavesdropping on the conversation cannot get any information.

### Performance Analysis

#### The Time Complexity of CP-ABE

In the setup phase, a global public key and a master key are generated. In this process, for each attribute *a*_*ij*_ in attribute space A, the attribute authority needs to compute Ψ_*ij*_. So the total time complexity of **Setup** is $O(\sum_{i=1}^{n}|A_{i}|)$ ($A_{i}\in A$).

When generating the private key for a user with attribute list *L*, the attribute authority needs to run |*L*| addition operations in order to compute *D*_1_, so the time complexity of algorithm **KeyGeneration** is *O*(|*L*|).

In algorithm **Encrypt**, in order to compute *C*_1_, the user needs to run |*W*| multiplication operations. So the time complexity is *O*(|*W*|).

In algorithm **Decrypt**, only two bilinear operations are needed, so the time complexity is *O*(1).

#### The Time Complexity of FastFilter

The algorithm **FastFilter** contains two main steps. In the first step, H is generated, which needs |*R*||*L*| loops. So the time complexity is *O*(|*R*||*L*|). In the second step, function **Combine** is called to generated $L_{can}$. In the worst case, the number of loops in **Combine** is $\prod_{i=1}^{\left|H\right|}\left|H_{i}\right|$. Therefore, the worst time complexity of **FastFilter** is $O(|R||L|+\prod_{i=1}^{\left|H\right|}|H_{i}|)$. Prime *λ* is an important parameter for **FastFilter**. Given a user with attribute list *L* and a remind vector *R*, let *q* = |*R*| and *p* = |*L*|, the probability of this user being ruled out before the second step (lines 15–16 in algorithm 1) is 1 − (1 − (1 − 1/*λ*)^*p*^)^*q*^, and the expected value of *H*_*i*_ is *p*/*λ*. From these two formulas, we can easily see that the larger the *λ*, the higher the efficiency. Meanwhile, the larger *λ* will weaken the security due to the decreased difficulty of dictionary profiling. Therefore, choosing an appropriate value for *λ* is the key to achieving a balance between security and efficiency.

#### The Time Complexity of CipherDecryption

The final matching step is done by algorithm **CipherDecryption**. For each $L_{can}^{i}\in L_{can}$, it calls **KeyGenU** to generate a candidate private key, then calls **Decrypt** to decrypt the ciphertext. Since the time complexity of **Decrypt** is *O*(1), the time complexity of **CipherDecryption** is $O(|L_{can}|)$.

## Simulation and Evaluation

We implement our protocol prototype on a laptop, which has Intel CORE i5 2.30 GHz Processor, 2GB RAM, Ubuntu 14.04 OS and Python 3.4.3. Thanks to CHARM [23], which is a python framework for rapidly prototyping advanced cryptosystems, we can easily implement the CP-ABE algorithm. In our experiment, we, firstly, test the performance of the CP-ABE algorithms. Secondly, we test the performance of FastFilter under different conditions. Finally, we test the performance of the complete profile matching protocol.

### The Performance of CP-ABE

**Fig 4:**
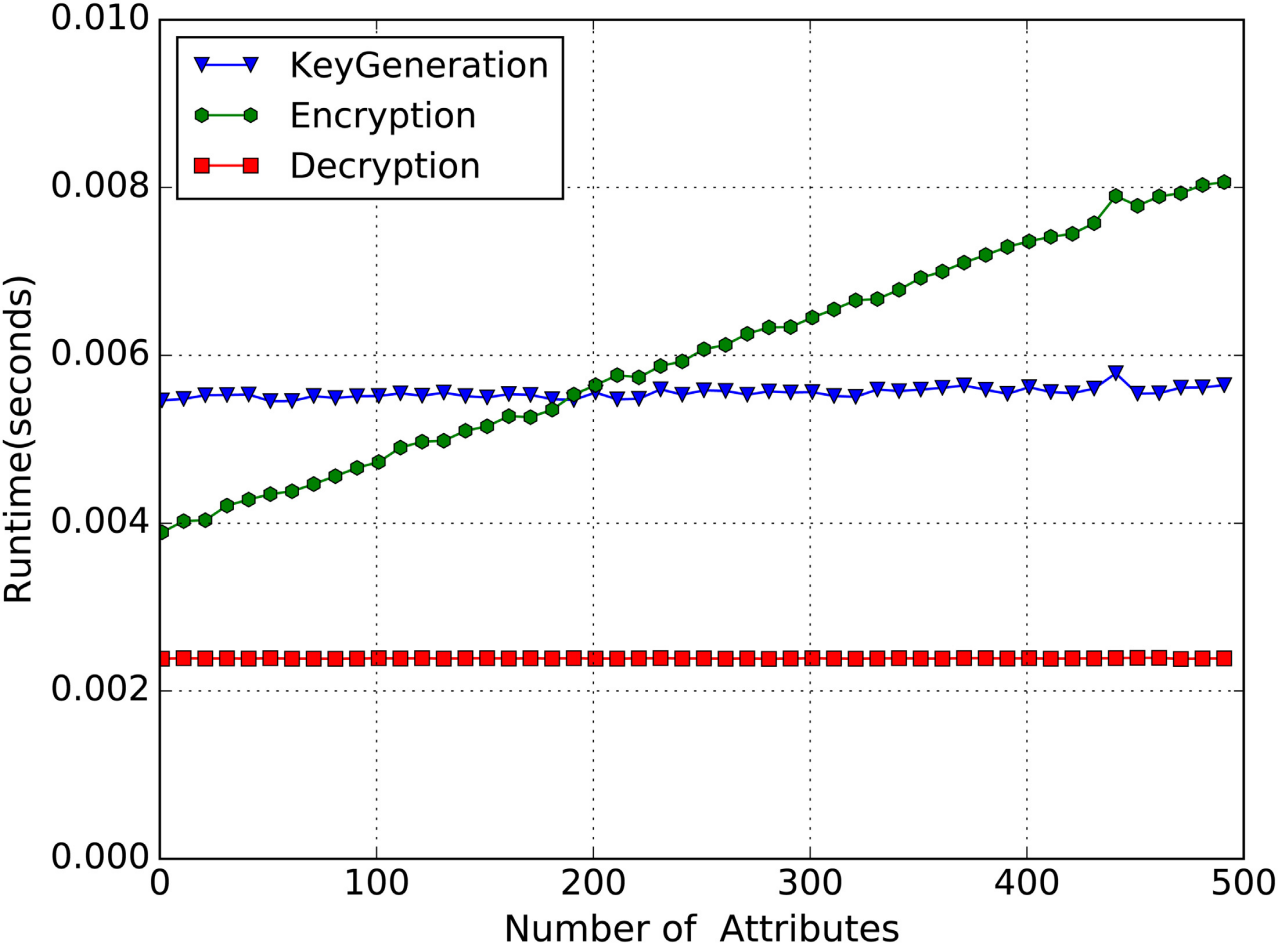
The performance of CP-ABE.

In order to evaluate the performance of our CP-ABE construction, we test the runtime of the core algorithms **KeyGeneration**, **Encryt** and **Decrypt** with different number of attributes (from 1 to 500). Fig 4 shows the test result. We can clearly see from Fig 4 that the key generation time and the encryption time increase with the number of attributes linearly, and the decryption time keeps constant. This result is in agreement with our time complexity analysis in section Security and Performance Analysis.

### The Performence of FastFilter

It is hard to figure out the precise time complexity of algorithm **FastFilter** theoretically. According to our previous analysis, the efficiency of **FastFilter** is correlated with the parameter *λ* and the length of query attribute list. We choose the number of loops in function **Combine** (lines 14, 15) as the performance indicator and design an experiment to evaluate the practical efficiency.

**Fig 5:**
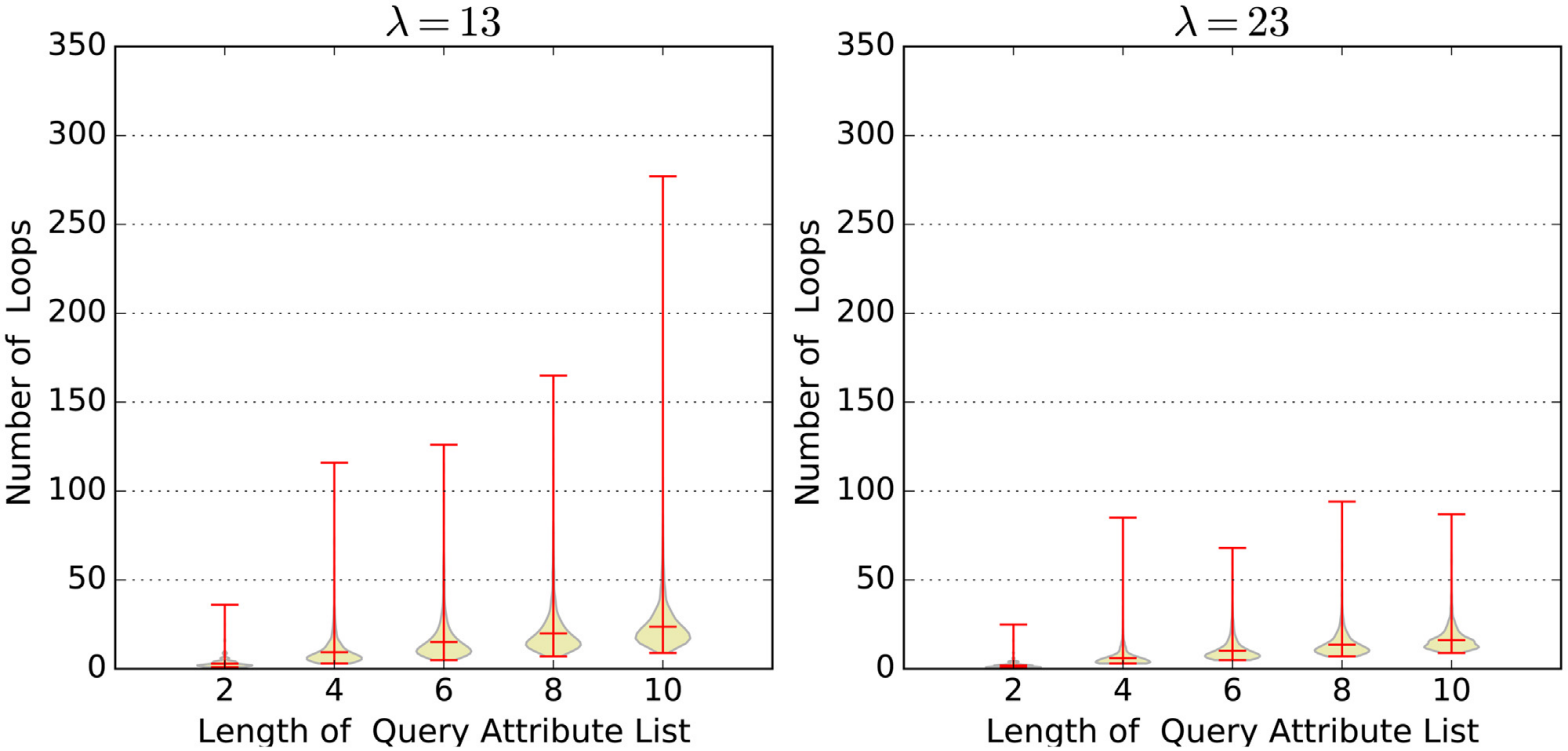
The number of loops in funciton Combine.

**Fig 6:**
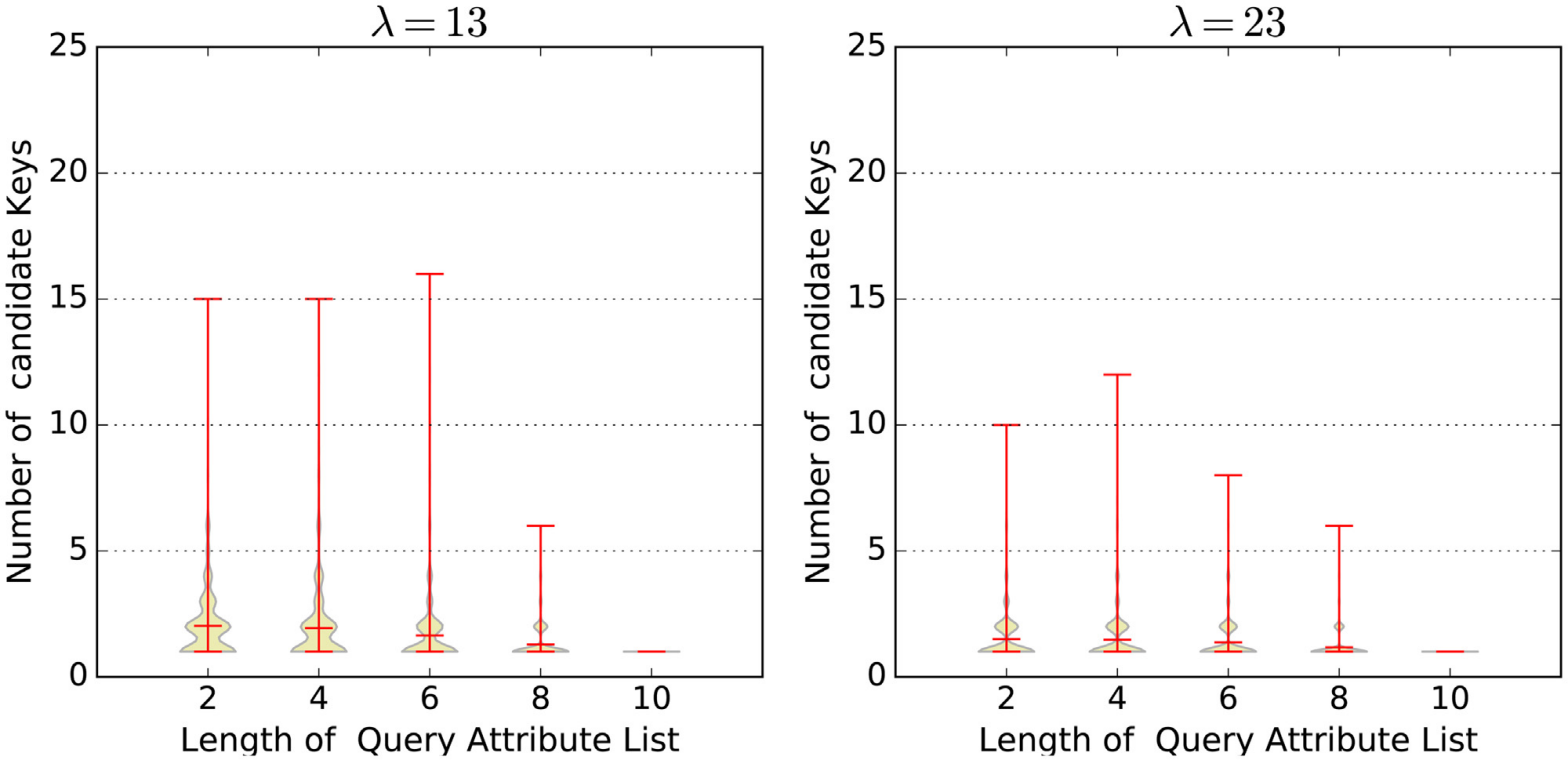
The number of candidate keys.

In our experiment, we assume the length of the user attribute list is 10. We choose different values for *λ* and *q*. Here, *q* is the length of the query attribute list. For each value combination (*λ*, *q*), we randomly generate 2000 user attribute lists and 2000 correspongding query attribute lists, run algorithm **FastFilter**, and record the number of loops in **Combine**. Finally, we draw the violin plots to show the value distribution. Fig 5 shows the test results, from which we can see three facts clearly. First, the mean values under different (*λ*, *q*) condations are all smaller than 25. Most of the results are distributed around the mean value. This fact implies the actual time complexity of **FastFilter** is far less than $O(|R||L|+\prod_{i=1}^{\left|H\right|}|H_{i}|)$. Second, larger *λ* can significantly reduce the number of loops (especially the max value), and thus improve the algorithm performance. Third, given *λ*, the larger the *q*, the more the number of loops. These two facts confirm our intuition. Since the time complexity of **CipherDecrytion** is $O(|L_{can}|)$, we also record the length of $L_{can}$ for each simulation in our experiment. The distribution can be seen in Fig 6.

### Integrated Test

**Fig 7:**
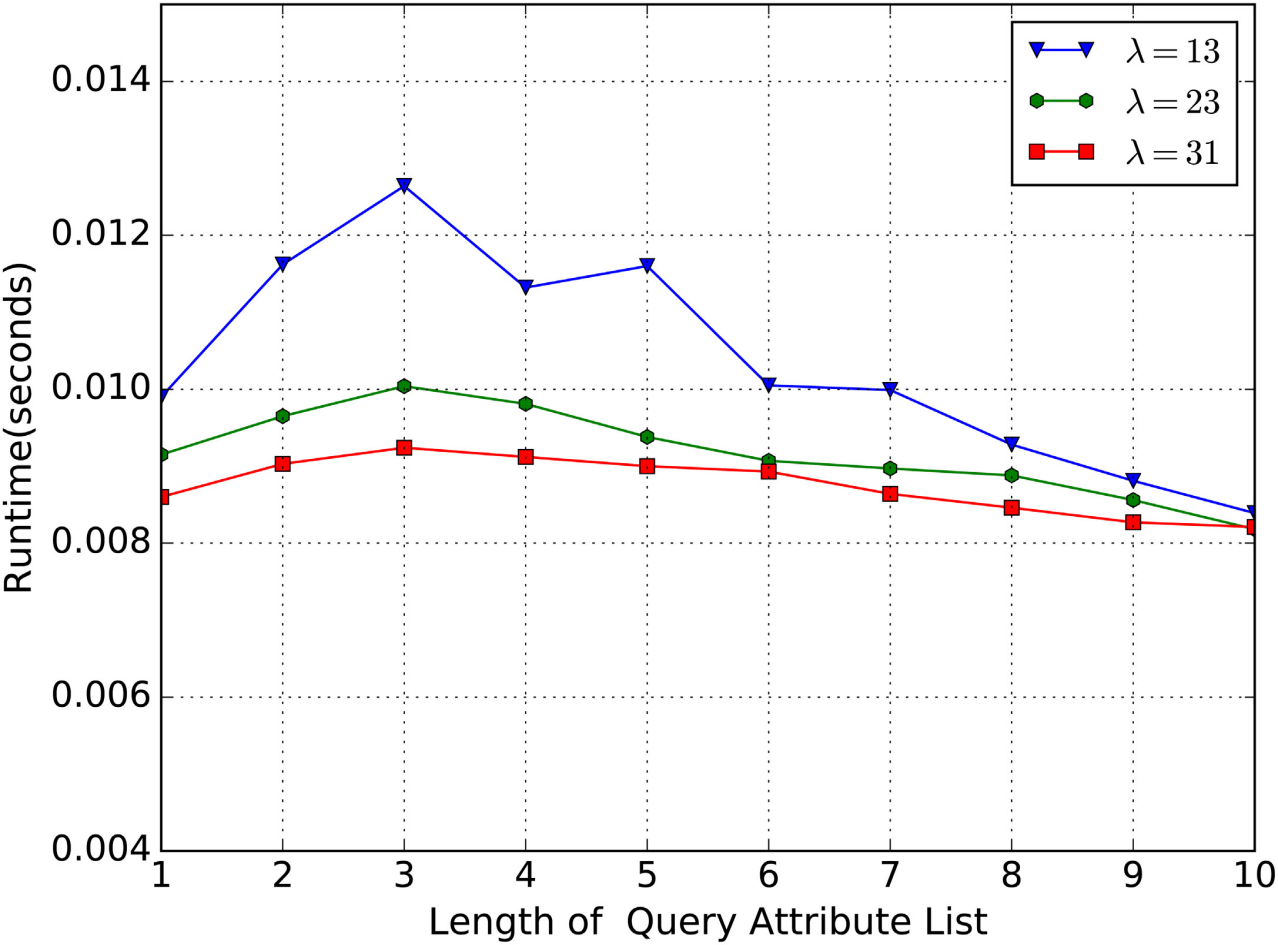
The integrated test result.

In order to evaluate the performance of the complete profile matching protocol, we test the time cost from a user beginning to run **FastFilter** until ultimately successful matching under different conditions. In our experiment, the length of user attribute list is assumed to be 10. Fig 7 shows the test result, from which we can clearly see the change of the runtime under different (*λ*, *q*). The test result is consistent with our anlaysis.

## Related Works and Comparison

### Related Works

#### Privacy-Preserving Profile Matching

In this subsection, we give a brief introduction of five related privacy-preserving profile matching schemes. Ming Li *et al.* in [9] proposed FindU (referred to as *A* hereafter), a set of privacy-preserving profile matching schemes for proximity-based MSN. In their schemes, each user own a set of attributes just like ours. An initiator searches for the best matched user by calculating the intersection of attributes between him/her and other users. For privacy preserving, FindU leverages secure multi-party computation (SMC) techniques to achieve PSI and PSCI.

Rui Zhang *et al.* in [13] proposed schemes (referred to as *B* hereafter) that can achieve fine-grained private matching for MSN. Their solutions feature fine-grained personal profiles in which each attribute is associated with a user-specific integer value indicating the corresponding user’s association with this attribute. The matching relationship between two users is determined by the similarity degree of their attribute vectors. Relating the computation of the similarity degree of two attribute vectors to secure dot-product computation (SDC) based on homomorphic encryption is their basic idea for privacy preserving.

The scheme (referred to as *C* hereafter) proposed by Vacha Dave *et al.* in [11] is also based on secure dot-product computation by using homomorphic encryption. In contrast to *B*, the user attribute vector in this scheme just consists of a number of specific attributes, which is coarser than the attribute vector in the scheme *B*. A distinctive feature of the scheme *C* is that it provides both privacy and verifiability, which means any participant cannot falsify the comparison result. To achieve this goal, each side of a matching pair has to compute the dot-product of their attribute vectors by using homomorphic encryption, which is computation expensive in practice.

Lan Zhang *et al.* in [24] proposed a more lightweight privacy-preserving profile matching scheme (referred to as *D* hereafter). In contrast to these schemes mentioned above, *D* does not use any SMC techniques. It leverages the fact that the intersection of the request profile and the matching profile is a nature common secret shared by the initiator and the matched user. Its main idea is to use the request profile (a set of attributes) as a key to encrypt a message. Only the matched user, who shares the secret, can decrypt the message efficiently.

The scheme (referred to as *E* hereafter) which is the most similar to ours was proposed by Hongjuan Li *et al.* in [25]. It is also based on CP-ABE. In contrast, the CP-ABE construction used in this scheme is not receiver anonymous and Bloom filter is used for access policy hiding and fast filtering.

#### Establishing Secure Channel

The establishment of a secure communication channel is always a hot topic in network security research. Especially in recent years, a lot of authentication and key exchange protocols have been proposed for various kinds of wireless networks to establish secure communication channels between their entities. For example, literature [26] presented a smart-card based user authentication scheme for Wireless Sensor Networks. Literature [27] presented an authenticated key exchange protocol for Wireless Body Area Network. Literature [28] presented an authentication and key exchange protocol for Vehicular Networks. Although we mainly focus on privacy preserving profile matching in this paper, using our scheme, the shared key can be exchanged between the matched pair securely and authentically. Based on this, we will try to design an attribute based authentication and key exchange protocol and apply it in the wireless networks mentioned above in our further research.

### Comparison

In this subsection, we compare our scheme with the five profile matching schemes mentioned above in terms of security, efficiency and usability.

#### Security

For the schemes *A*, *B*, the main security problem is the lack of verifiability. In the scheme *A*, the matching computation between two users needs assistance from extra 2*t* − 2 users. Due to the lack of verifiability, any assistant user can falsify the computation result so as to cause a wrong matching result. In the scheme *B*, it is also easy for an adversary to cheat the initiator with a false dot-product result. Although the scheme *C* provides verifiability, it needs each side of a matching pair to compute the dot-product of their attribute vectors by using homomorphic encryption, which means larger computation overhead. For the scheme *D*, due to the lack of the authentication of user’s attributes, any user can arbitrarily change their attribute lists locally and quickly. Therefore, the scheme *D* can not well defend the dictionary attack and collusion attack. In the scheme *E*, Bloom filter is used for access policy hiding and fast filtering. However, different from our reminder vector which only gives out a fuzzy hint about the preference profile, the adversary can determine that whether or not an attribute is in the preference-profile accurately duo to the low false-positive probability of Bloom filter. Therefore, the scheme *E* is also unable to defend the dictionary attack. Compared with these five schemes, our scheme provides implicit verifiability which do not need extra computation cost. Due to the security features of anonymous CP-ABE and reminder vector, our scheme can well defend the dictionary attack and collusion attack. In addition, our scheme can establish a secure communication channel between the pair of matched users in a natural way, which is ignored by schemes *A*, *B* and *C*.

#### Efficiency

For the schemes A, B and C, the matching result is determined by the initiator ultimately. In other words, the initiator has to further compute and make a decision according to the computation results sent back from other participants. When the number of participants becomes lager, the initiator may face a lager computation burden which will decrease the computation efficiency and increase the power consumption. In addition, whether or not matching the initiator’s profile, all participants have to take on nearly the same computation overhead and interact with the initiator at least two times. It is a reasonable inference that the overall network performance will be greatly affected when the number of initiators is larger. In contrast, in our scheme, the matching result can be determined by the users who receive the query package rather than the initiator. For a given initiator, most of the participants will be ruled out in the fast filtering phase quickly. Only a few candidates need to further generate the private keys and try to decrypt the ciphertext. That is why our scheme is more efficient.

#### Usability

In the first four schemes (A, B, C and D), users have to define a security parameter called threshold which directly affects the matching result. In our opinion, it is difficult for a normal user to intuitively understand the meaning of the threshold and assign value to it properly, which decreases the usability to a certain degree. In contrast, the only adjustable parameter in our scheme is *λ*, which is used to balance the security and efficiency. The value of *λ* has no affection on the matching result. Therefore, it can be preset as an default value. Normal users need not to care about it.

Table 3 summarizes the result of the comparison. Here, “Interaction Times” represents the communication times required for a successful profile matching between a pair of users, “Decider” represents the one who determines the profile matching result ultimately, and “Filterability” represents the scheme can provide a mechanism to fast exclude the unmatched users, which is significant for enhancing the overall performance.

**Table 3:** Comparison with related schemes.

Scheme	Key Idea	Interaction Times	Verifiability	Defending Dictionary Attack	Decider	Filterability
*A* [9]	PSI, PSCI	10(2*t* − 2)	No	No	Initiator	No
*B* [13]	SDC	2	No	No	Initiator	No
*C* [11]	SDC	5	Yes	No	Initiator	Yes
*D* [24]	SDC	2	Yes	No	Initiator	Yes
*E* [25]	CP-ABE	2	Yes	No	Initiator	Yes
Ours	CP-ABE	1	Yes	Yes	Matching User	Yes

## Conclusion

In this paper, we design a novel CP-ABE based privacy-preserving profile matching scheme for MSN. As far as we know, it is the first time to use CP-ABE technique to address the privacy-preserving profile matching problem. Our scheme is based on a special CP-ABE construction which provides receiver anonymity. The length of ciphertext and the number of pairing computations are all constant. Meanwhile, we design a fast filter algorithm to make the original CP-ABE construction applicable in our application context and improve the matching efficiency. The theoretical analysis and experimental results show the security and the better efficiency of our scheme. Finally, we compare our scheme with other four related state-of-the-art solutions, which shows the distinctive features and advantages of our scheme in terms of security, efficiency and usability. Our scheme is also applicable to many scenarios beyond the motivation problem in this paper, for example, the patient matching in online healthcare social networks.

## Supporting Information

S1 Appendix (PDF)
