# Supplementary material for: CP-ABE Based Privacy-Preserving User Profile Matching in Mobile Social Networks
Source: PLoS One. 2016 Jun 23;11(6):e0157933. doi: 10.1371/journal.pone.0157933 (PMC4919042; doi:10.1371/journal.pone.0157933)
Supplement: S1 Appendix — (PDF) [file pone.0157933.s001.pdf]

S1 Appendix

Proof of the Theorem 2

The construction of the simulator mentioned in theorem 1 is described as follows:

Setup. The simulator first sets an integer $u = 4q$, chooses the integer $k \in_R \{0, 1, 2, ..., n\}(n = |\mathbb{A}|)$, integer x', $x_{ij} \in_R \{0, 1, ..., u-1\}$. Meanwhile, it chooses y' , $y_{ij} \in_R Z_P^*$, $1 \leq i \leq n$, $1 \leq j \leq m$. All these parameters are kept by the simulator itself. For an attribute list $L = \{l_1, l_2, ..., l_q\}$, we define $F(L) = (p - uk) + x' + \sum_{a_{ij} \in L} x_{ij}$, $J(L) = y' + \sum_{a_{ij} \in L} y_{ij}$, and

$$K(L) = \begin{cases} 0, & x' + \sum\limits_{a_{ij} \in L} x_{ij} \equiv 0 \ (mod\ u) \\ 1, & otherwise. \end{cases}$$

For a given asymmetric DBDH tuple $(g, g^b, g^c, \hat{g}, \hat{g}^a, \hat{g}^b, Z)$, the simulator sets $\hat{g}_1 = \hat{g}^a$, $\hat{g}_2 = \hat{g}^b$, $g_2 = g^b$. Then the public parameter $\hat{f} = \hat{g}_2^{p-uk+x'} \hat{g}^{y'}$, $f = g_2^{p-uk+x'} g^{y'}$, $\Psi_{ij} = g_2^{x_{ij}} g^{y_{ij}}$. The Ψ_{ij} implies $\psi_{ij} = bx_{ij} + y_{ij}$.

Phase 1. The adversary $\mathcal{A}$ will issue private key queries. Suppose $\mathcal{A}$ issues a query for an private key associated with attribute list L. if $K(L) = 0$ simulator aborts and randomly chooses its guess $\beta^{'}$ of the challenger's value β. Otherwise, it chooses $r \in_R Z_p^*$, and computes

$$D_0 = \hat{g}_1^{-1/F(L)} \hat{g}^r, D_1 = \hat{g}_1^{-J(L)/F(L)} (\hat{f} \cdot \hat{g}_2^{\sum_{a_{ij} \in L} x_{ij}} \hat{g}^{\sum_{a_{ij} \in L} y_{ij}})^r.$$

Let $\tilde{r} = r - a/F(L)$, we can get

$$\begin{aligned} D_0 &= \hat{g}_1^{-1/F(L)} \hat{g}^r = \hat{g}^{r-a/F(L)} = \hat{g}^{\tilde{r}} \\ D_1 &= \hat{g}_1^{-J(L)/F(L)} (\hat{f} \cdot \hat{g}_2^{\sum_{a_{ij} \in L} x_{ij}} \hat{g}^{\sum_{a_{ij} \in L} y_{ij}})^r \\ &= \hat{g}^{ab-ab-aJ(L)/F(L)} (\hat{g}^{b(p-uk+x')+y'} \hat{g}^{\sum_{a_{ij} \in L} bx_{ij}+y_{ij}})^r \\ &= \hat{g}^{ab} (\hat{g}^{bF(L)} \hat{g}^{J(L)})^{-a/F(L)} (\hat{g}^{bF(L)} \hat{g}^{J(L)})^r \\ &= \hat{g}_2^a (\hat{g}_2^{F(L)} \hat{g}^{J(L)})^{r-a/F(L)} \\ &= \hat{g}_2^a (\hat{f} \hat{g}^{\sum_{a_{ij} \in L} \psi_{ij}})^{\hat{r}}. \end{aligned}$$

This simulator will be able to perform this computation iff $F(L) \neq 0\ mod\ p$. For ease of analysis, the simulator will only continue (not abort) in the sufficient condition where $K(L) \neq 0$ (If we have $K(L) \neq 0$ this implies $F(L) \neq 0\ mod\ p$ since we can assume $p > nm$ for any reasonable values of p, n, and m).

Challenge. The adversary $\mathcal{A}$ next will submit two equal length messages M_0, M_1 and two access policies W_0, W_1. The simulator flips two fair binary coin r and e. If $x' + \sum_{a_{ij} \in W_r} x_{ij} \neq uk$, the simulator will abort and give a random guess $\beta^{'}$. Otherwise, $F(W_r) \equiv 0\ (mod\ p)$, and the simulator outputs the ciphertext $T_c = \{ZM_r, C_0, C_0^{J(W_r)}\}$, in that $C_0 = g^c$,

$$\begin{aligned} C_0^{J(W_r)} &= g^{c(y' + \sum_{a_{ij} \in W_r} y_{ij})} \\ &= g^{c(y' + \sum_{a_{ij} \in W_r} y_{ij} + b(p - uk + x' + \sum_{a_{ij} \in W_r} x_{ij}))} \\ &= (g^{b(p-uk+x')} g^{y'} g^{b \sum_{a_{ij} \in W_r} x_{ij}} g^{\sum_{a_{ij} \in W_r} y_{ij}})^c \\ &= (f \cdot g^{\sum_{a_{ij} \in W_r} \psi_{ij}})^c \\ &= (f \prod_{a_{ij} \in W_r} \Psi_{ij})^c. \end{aligned}$$

Clearly, if $Z = e(g, \hat{g})^{abc}$, the T_c is a valid encryption of M associated with W_r. Otherwise, Z just is a random element from G_T, and the adversary is impossible to get any information about r and e.

Phase 2. The adversary $\mathcal{A}$ continues to issue private key queries with the requirement that the attribute list can not satisfy W_0, W_1. The simulator repeats the same method used in Phase 1.

Guess. The adversary $\mathcal{A}$ outputs a guess (r', e') of (r, e)

Artificial Abort. If the simulator has not aborted at this point it will check to see if the adversary's guess $r = r'$ and $e = e'$. If $r' = r$ and $e = e'$ then the simulator outputs a guess $\beta' = 1$, otherwise it outputs $\beta = 0$.

Intuitively, under the condition of $Z = e(g, \hat{g})^{abc}$, if $\mathcal{A}$ can always successfully guess r' within a probability polynomial time, it means the simulator can resolve the asymmetric DBDH problem within a probability polynomial time as well, which contradicts to the assumption that the asymmetric DBDH is NP-hard. This simulator is difficult to be analyzed directly since it might abort before all of the queries are made. The key point is that we should figure out an appropriate estimate of the simulator abort probability because $Pr[\beta' = \beta] = Pr[\beta' = \beta | abort] Pr[abort] + Pr[\beta' = \beta | \overline{abort}] Pr[\overline{abort}]$. Hereafter, we adopt the same proof method as in literature [22] and give out the same conclusion mentioned in theorem 1. Since the complete proof process is longer, we omit it here due to space limitation and direct the interested reader to [22].
